# Frequency of acute kidney injury in post-liver transplantation and associated factors: a systematic review

**DOI:** 10.1590/2175-8239-JBN-2025-0022en

**Published:** 2025-10-10

**Authors:** Ana Flavia Moura, Alessandra Lima Costa, Maria Theresa Corrêa Evangelista, Ana Clara de Lemos Guimarães, Arthur Guimarães de Freitas, Gabriel Pla Cid Vinhaes, Maria Eduarda Serravalle Mata Pires Fernandes, Daniela de Queiroz Moura-Landim, José A. Moura-Neto, Constança Margarida Sampaio Cruz

**Affiliations:** 1 Escola Bahiana de Medicina e Saúde Pública, Departamento de Medicina Interna, Salvador, BA, Brazil.; 2 Universidade Federal da Bahia, Salvador, BA, Brazil.; 3 Universidade de Salvador, Salvador, BA, Brazil.; 4 Hospital Santo Antônio – Obras Sociais Irmã Dulce, Salvador, BA, Brazil.

**Keywords:** Acute Kidney Injury, Kidney Failure, Liver Transplantation, Systematic Review

## Abstract

**Introduction:**

Acute kidney injury (AKI) is a common complication following liver transplantation (LT). It is associated with factors such as perioperative hemodynamic instability, prolonged surgery, and use of nephrotoxic immunosuppressants, contributing to increased mortality, graft failure, and extended hospital stay.

**Methods:**

A systematic search of the databases PubMed, Embase, and the Cochrane Central Register of Controlled Trials was conducted to identify observational studies with samples of at least 50 patients aged 18 years or older who underwent LT and analyzed AKI incidence post-procedure and assess long-term renal outcomes.

**Results:**

A total of 30 studies with a total of 13,653 patients were included. The incidence of AKI post-LT was 46% (95% CI: 45%–47%), with significant variation across studies (24% to 84%) and high heterogeneity (I^2^ = 97%, p < 0.001). The pooled incidence of dialysis requirement post-LT was 10% (95% CI: 9%–11%), also highly variable across studies (2% to 36%) with high heterogeneity (I^2^ = 95%, p < 0.001). Common postoperative complications included prolonged mechanical ventilation, graft dysfunction, infections, and hypertension (HTN). Furthermore, the analysis highlighted significant AKI risk factors, such as HTN, diabetes, hyperlactatemia, hyperbilirubinemia, and prolonged hospitalization.

**Conclusion:**

AKI and dialysis requirements are frequent complications following LT. Multiple risk factors, including HTN, diabetes, and prolonged hospitalization, are associated with an increased risk of AKI post-LT. The high incidence of AKI underscores the importance of early identification of at-risk patients and multidisciplinary approaches to improve outcomes.

## Introduction

Acute kidney injury (AKI) is a clinical syndrome characterized by a rapid decline in the glomerular filtration rate, leading to the accumulation of metabolic products and an increased risk of mortality, cardiovascular events, and progression to chronic kidney disease (CKD)^1,2^. AKI incidence is particularly high in patients undergoing liver transplantation (LT), affecting 30% to 40% of recipients within the first postoperative week. This condition is influenced by several factors, including preoperative conditions, perioperative hemodynamic instability, prolonged surgery time, and the use of nephrotoxic immunosuppressive drugs^3,4,5^.

AKI is a common and concerning complication in the postoperative period of LT due to its association with poor prognosis. Previous meta-analyses have shown that AKI after LT is linked to higher mortality, graft rejection, and CKD progression. Identified risk factors include vasopressor use, blood transfusions and pre-existing liver dysfunction. However, these studies lack recent data, limiting the current evidence on this topic^3,6^. Additionally, AKI prolongs hospitalization and increases treatment costs, making its management a priority^7,8,9^. Thus, a multidisciplinary and preventive approach can significantly reduce complications and improve the prognosis of liver transplant patients.

The complexity of the association between LT and renal function underscores the need for a careful management of these patients. According to recent AKI guidelines, both the *Kidney Disease: Improving Global Outcomes* (KDIGO)^10^ and the *International Society of Nephrology* (ISN)11 emphasize the importance of early risk identification and stratification for AKI, particularly in patients undergoing major surgery, experiencing sepsis, or exposed to nephrotoxins. Preventive strategies include ensuring adequate hydration with goal-directed fluid therapy, avoiding both hypovolemia and fluid overload and minimizing the use of nephrotoxic agents such as aminoglycosides, non-steroidal anti-inflammatory drugs, and iodinated contrast^10,11^. Management and prevention combined with advances in surgical techniques and intensive care have the potential to significantly reduce complications and improve long-term outcomes for transplant recipients^10,12^.

Given this context, the objective of this meta-analysis is to compile detailed data from studies to evaluate the incidence of AKI post-LT and assess long-term renal outcomes, such as CKD progression and dialysis dependency. This will serve to optimize prevention and treatment strategies, thereby improving clinical outcomes and the quality of life of transplanted patients.

## Method

### Study Design

This is a systematic review and meta-analysis examining the incidence of AKI after LT surgery and associated factors, including related disorders, need for dialysis treatment, and patient age. The study was registered on the PROSPERO platform (International Prospective Register of Systematic Reviews) under the code CRD42024583773.

### Search Strategy

A comprehensive search of the literature was conducted in both Portuguese and English, covering all available studies up to July 2024. Searches were performed in PubMed, Cochrane, and Embase databases using health-related descriptors from DeCS/MeSH platforms, combined with Boolean operators “OR” and “AND” to target the population of interest. Specifically, the following terms were used: “(LT OR OLT OR liver transplantation OR Grafting, Liver OR Liver Grafting OR Transplantation, Liver OR Liver Transplantations OR Liver Transplant OR Liver Transplants OR Transplant, Liver OR Hepatic Transplantation OR Hepatic Transplantations OR Transplantation, Hepatic) AND (AKI OR acute kidney injury OR Acute Kidney Injuries OR Kidney Injuries, Acute OR Kidney Injury, Acute OR Acute Renal Injury OR Acute Renal Injuries OR Renal Injuries, Acute OR Renal Injury, Acute OR Renal Insufficiency, Acute OR Acute Renal Insufficiencies OR Acute Kidney Failure)”.

### Inclusion Criteria

The review included prospective and retrospective observational studies with a sample size of at least 50 patients with at least 18 years of age undergoing LT surgery, which analyzed AKI incidence post-procedure.

### Exclusion Criteria

Studies were excluded if they involved populations under 18 years of age, chronic or pre-existing AKI cases, reviews (narrative, scoping, integrative, or systematic), case reports/series, conference abstracts, incomplete or overlapping data, or were not in Portuguese or English. Interventional studies were also excluded.

### Data Selection and Extraction

Articles were identified based on the search strategy and imported into Rayyan software. Duplicate entries were removed using both the software and manual cross-checks. Article selection was performed manually by two independent researchers in a blinded manner; discrepancies were resolved by a third investigator. Selected studies were organized in Microsoft Excel, where data such as publication year, country, sample characteristics (size, age and gender), AKI incidence post-LT, AKI definitions, main outcomes, risk of bias, dialysis requirement and associated risk factors were extracted.

### Quality Assessment

The methodological quality of the included studies was evaluated using the Newcastle-Ottawa Quality Assessment Form for Cohort Studies.

### Systematic Review and Meta-Analysis

A systematic literature review was conducted, followed by a meta-analysis of AKI incidence and the need for dialysis treatment post-LT through data interpolation.

## Results

### Selection and Characterization of the Selected Studies

**Figure 1:**
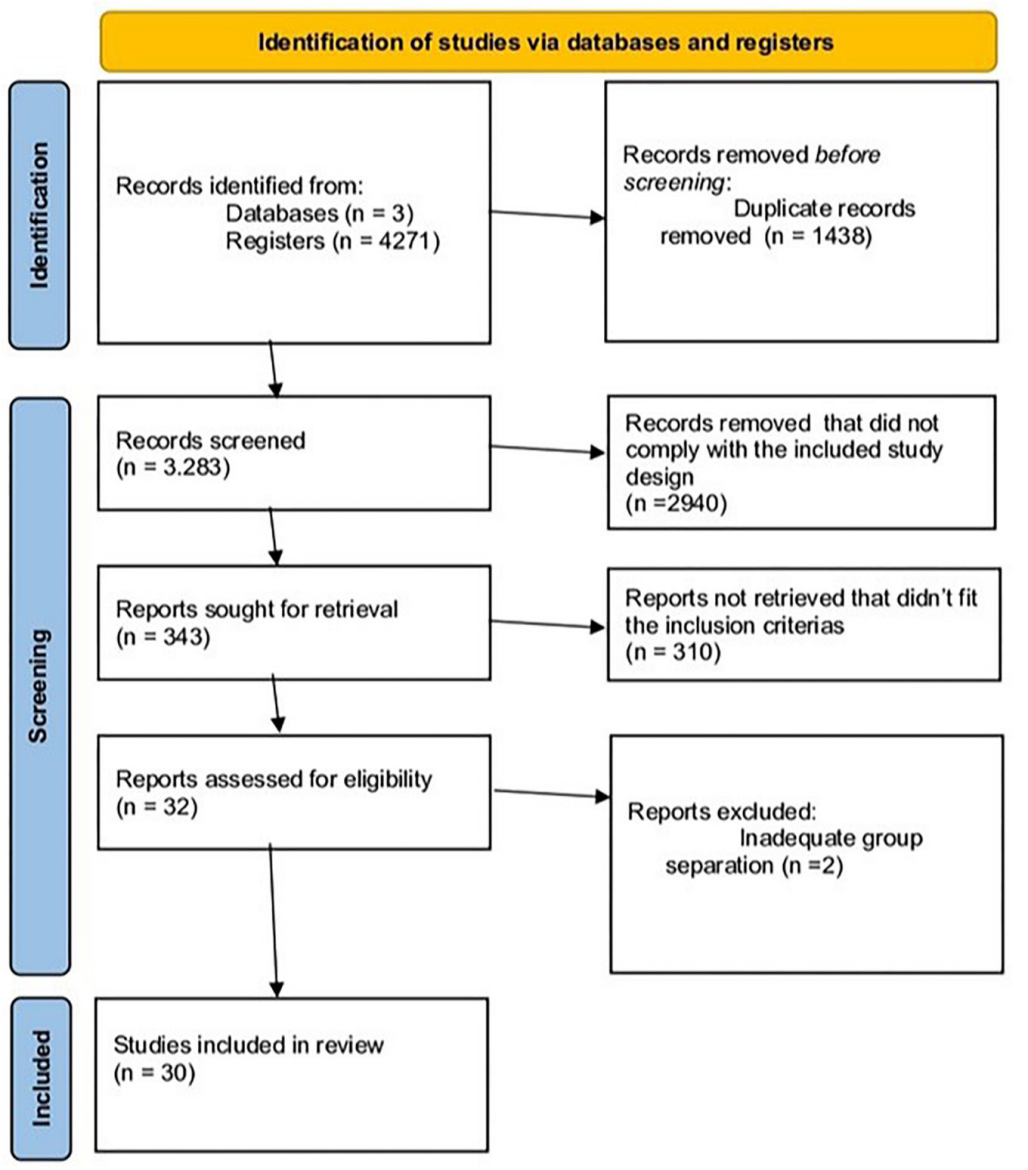
PRISMA 2020 flow diagram for systematic reviews, which included searches of databases and registers only.

From the systematic search and application of inclusion and exclusion criteria, 30 articles were included, involving a total of 13,653 patients. The PRISMA flowchart is shown in Figure 1, and the risk of bias in Supplementary Table S1. Most studies were of high or moderate evidence quality, as evaluated using the Newcastle-Ottawa Quality Assessment Form for Cohort Studies.

The included studies were published between 2019 and 2024. Of the total number of patients, 6,122 developed AKI after LT surgery. All included studies were prospective or retrospective observational studies published in English, with one study (3.2%) being a case-control study (Table 1). Most studies utilized KDIGO guidelines for AKI definition, except five studies (16%) that used RIFLE or AKIN definitions (Table 2). Tables 1 and 2 summarize the characteristics of each individual study.

**Table 1:** General characteristics of the included studies

Author	Year	Country	Study design	Sample size	Age	Female
Cywinski et al^13^	2024	USA	Retrospective	1576	56 ± 11	33%
Caragata et al^14^	2023	Canada	Retrospective	1292	58 ± 18	68%
Chiu et al^15^	2023	Taiwan	Retrospective	392	54 ± 8	23.7%
Rana et al^16^	2023	India	Prospective	186	not available	68%
Cai et al^17^	2023	China	Retrospective	214	45 ± 11	13%
Wu et al^18^	2023	China	Prospective	120	53 ± 10	20%
de la Fuente et al^19^	2022	Spain	Prospective	150	55.6 (51-63)	23%
Berkowitz et al^20^	2022	USA	Retrospective	598	54 ± 11	34%
Chan et al^21^	2022	Taiwan	Retrospective	512	54 ± 8	26%
Fiorelli et al^22^	2022	Italy	Prospective	1681	53	25%
Catalán et al^23^	2022	Mexico	Retrospective	86	52 (18-69)	53%
Park et al^24^	2021	South Korea	Retrospective	591	53 ± 9	70%
Chen et al^25^	2021	China	Retrospective	1,505	49 ±10	18%
Zhang et al^26^	2021		Retrospective	204	49	20%
Park et al^27^	2020	South Korea	Retrospective	479	53 (48 - 59)	31%
Ren et al^28^	2020	China	Retrospective	173	51 ± 9	8%
Mrzljak et al^29^	2020	Croatia	Retrospective	205	57 ±10	26%
Savier et al^30^	2020	France	Retrospective	159	50	not available
Guo et al^31^	2020	China	Case Control	122	51 ± 11	20%
Shih et al^32^	2020		Retrospective	60	55 ± 6	not available
Carrier et al^33^	2020	Canada	Retrospective	524	52 ± 11	33%
Lee et al^34^	2020	South Korea	Retrospective	294	52,7	53%
Kim et al^35^	2020	South Korea	Retrospective	676	54,7	28%
Feldkamp et al^36^	2020	Germany	Retrospective	149	48,6	37%
Min et al^37^	2020	South Korea	Retrospective	423	51,5	26%
Kim et al^38^	2019	South Korea	Retrospective	734	53 (48-58)	24%
Tan et al^12^	2019	China	Retrospective	227	46 ± 10	19%
Arani et al^39^	2021	Iran	Retrospective	173	45 +- 17	45%
Neves et al^40^	2022	Brazil	Retrospective	49	54 (43-65)	39%
Bao et al^41^	2021	China	Retrospective	99	49 ± 10	8%

Abbreviations – Fem: Female. Categoric and numeric variables were described using mean and standard deviation; median and interquartile range; valid percentage or absolute value.

**Table 2:** Specific characteristics of the included studies

Author	Year	AKI definition	AKI incidence	AKI age	Female AKI	Dialysis
Cywinski et al^13^	2024	KDIGO	73.6%	not available	not available	not available
Caragata et al^14^	2023	KDIGO	40%	not available	not available	52
Chiu et al^15^	2023	KDIGO	62%	54 ± 8	25.3%	17
Rana et al^16^	2023	ni	31.7%	51 (41-57)	5.1%	25
Cai et al^17^	2023	KDIGO	49%	48 ± 10	11.4%	10
Wu et al^18^	2023	KDIGO	48.3%	53 (11)	22.4%	8
de la Fuente et al^19^	2022	KDIGO	58.6%	58 (51-65)	22%	51
Berkowitz et al^20^	2022	KDIGO	42.6%	52	33%	not available
Chan et al^21^	2022	KDIGO	35%	55 ± 8	27%	not available
Fiorelli et al^22^	2022	RIFLE	21.8%	54	26%	not available
Catalán et al^23^	2022	KDIGO	39.5%	51 ± 11	35.2%	9
Park et al^24^	2021	KDIGO	25.2%	53 (48-59)	23%	35
Chen et al^25^	2021	KDIGO	44.5%	49 ± 10	19%	not available
Zhang et al^26^	2021	KDIGO	55.3%	49	12%	not available
Park et al^27^	2020	KDIGO	24%	52 (47-59)	27%	not available
Ren et al^28^	2020	KDIGO	27.7%	52 ± 10	10%	23
Mrzljak et al^29^	2020	KDIGO	45.3%	56 ± 9	22.6%	5
Savier et al^30^	2020	KDIGO	29%	not available	not available	not available
Guo et al^31^	2020	KDIGO	42.6%	53 ± 11	29%	12
Shih et al^32^	2020	ni	41.6%	not available	not available	not available
Carrier et al^33^	2020	ni	69.8%	not available	not available	31
Lee et al^34^	2020	AKIN	25.8%	55	72%	not available
Kim et al^35^	2020	KDIGO	40.6%	not available	not available	not available
Feldkamp et al^34^	2020	KDIGO/ RIFLE / AKIN	65.7%	47	33%	54
Min et al^36^	2020	AKIN	87.2%	52	29%	not available
Kim et al^37^	2019	KDIGO	36.1%	54 (49-59)	26%	not available
Tan et al^12^	2019	KDIGO	46.6%	47 ± 10	15%	11
Arani et al^39^	2021	AKIN	68.2%	48 ±15	46%	25
Neves et al^40^	2022	KDIGO	83.6%	61 (43-65)	22%	not available
Bao et al^41^	2021	KDIGO	29.2%	50 ± 8	3.4%	4

Abbreviations – AKI: Acute kidney injury; KDIGO: Kidney Disease Improving Global Outcomes; AKIN: Acute Kidney Injury Network; RIFLE: Risk Injury Failure Loss End-stage kidney disease; Fem: Female. Categoric and numeric variables were described using mean and standard deviation; median and interquartile range; valid percentage or absolute value.

### Incidence of Aki After Liver Transplantation

**Figure 2:**
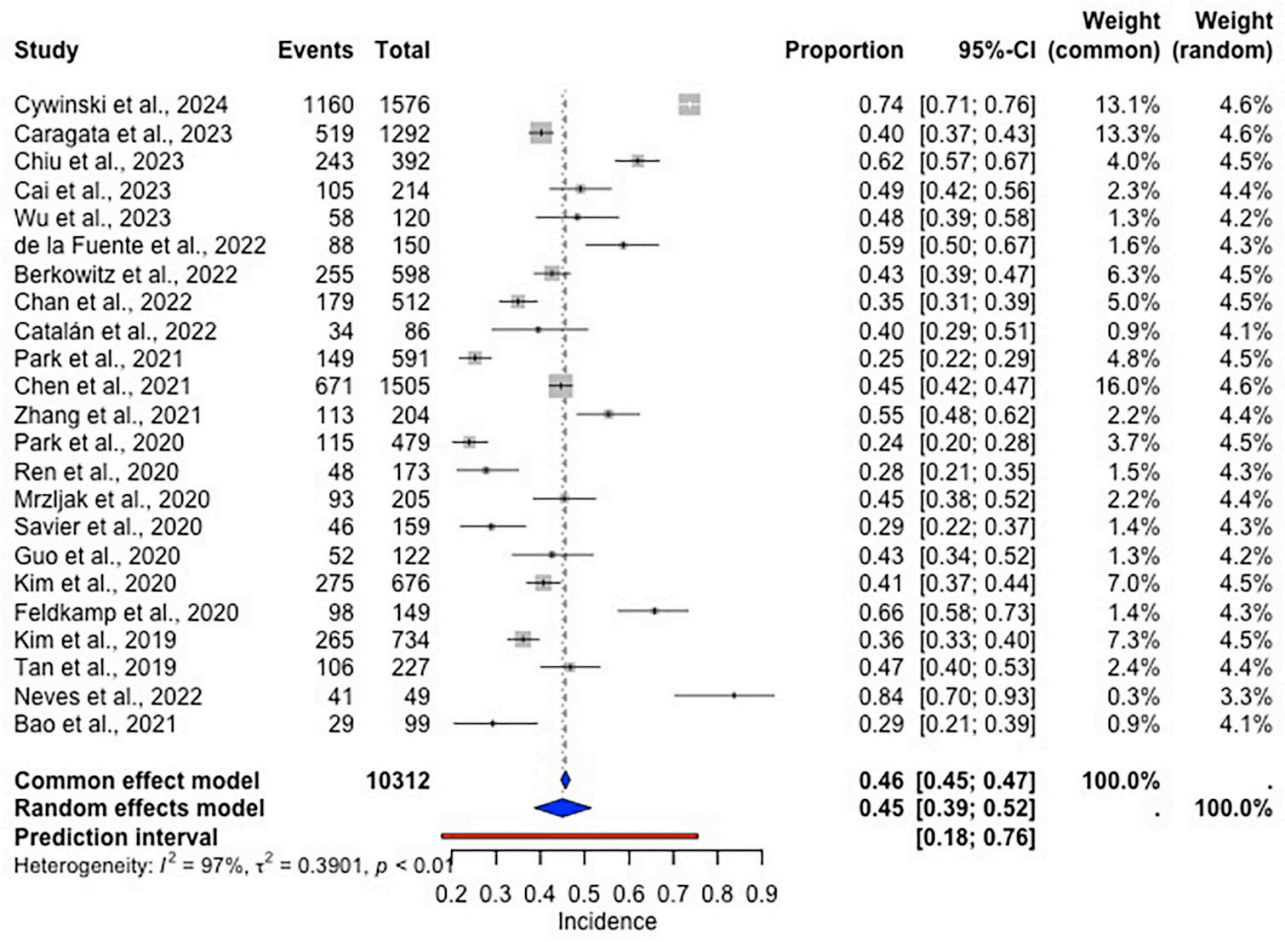
Forest plot of the estimated incidence of AKI after LT.

The estimated incidence of AKI after LT was 46% (95%CI: 45–47%, Figure 2). A sensitivity analysis was performed only with studies that used the KDIGO definitions for AKI.

### Dialysis Treatment for Aki After Liver Transplantation

**Figure 3:**
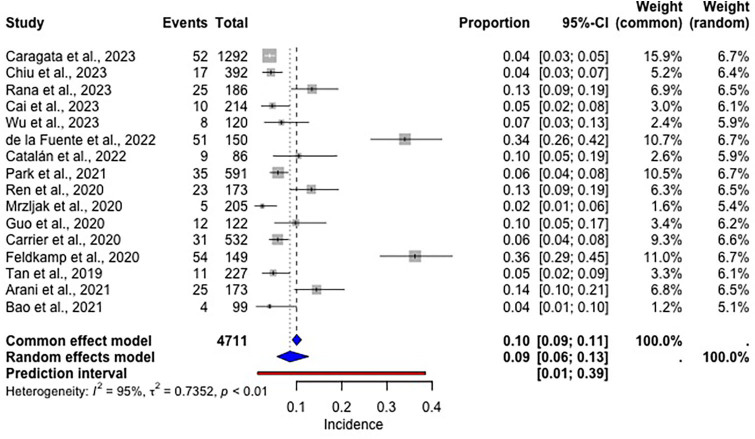
Forest plot of the estimated incidence of patients that required dialysis following liver transplantation.

A total of 423 patients required dialysis following liver transplantation, with an incidence of 10% (95% CI: 9–11%, Figure 3).

### Documented Disorders

Other documented disorders and complications following LT surgery are shown in Table 3, including prolonged mechanical ventilation, graft-related irregularities, infections, hypertension (HTN), and others.

**Table 3:** Other documented disorders and complications following lt surgery

Author	Year	Other documented disorders
Cywinski et al^13^	2024	Major cardiovascular events (MACE) and early graft dysfunction (EAD)
Caragata et al^14^	2023	Early mortality, cardiac complications, and early graft dysfunction
Chiu et al^15^	2023	Diabetes and hypertension
Rana et al^16^	2023	Chronic kidney disease
Cai et al^17^	2023	Infections (pulmonary and sepsis), gastrointestinal hemorrhage, stroke, and graft-versus-host disease (GVHD).
Wu et al^18^	2023	Postoperative infections, prolonged time on mechanical ventilation, postoperative shock, and early graft dysfunction
de la Fuente et al^19^	2022	Prolonged duration of mechanical ventilation, nosocomial infections, early hepatic graft dysfunction.
Fiorelli et al^22^	2022	Longer time on mechanical ventilation
Zhang et al^26^	2021	Hyperlactatemia
Savier et al^30^	2020	Biliary complications
Kim et al^35^	2019	Elevated baseline venous pressure, decreased oxygen saturation in venous blood, and increased right ventricle end-diastolic volume
Tan et al^12^	2019	Infections and prolonged intubation time
Neves et al^40^	2022	Need for vasopressor, high lactate, transfusion and reintervention
Bao et al^41^	2021	Hypertension

### Documented Risk Factors

Table 4 presents the risk factors for AKI after LT identified in the studies included in this meta-analysis. The risk of developing AKI was influenced by a range of non-modifiable preoperative factors, including demographic variables such as male sex and, in some cohorts, younger age. Clinical conditions such as diabetes mellitus, HTN, hepatic encephalopathy, and hepatocellular carcinoma contributed to an increased susceptibility. Baseline renal impairment (evident through elevated creatinine, proteinuria, or reduced glomerular filtration rate) further exacerbated risk. Laboratory abnormalities, including hypoalbuminemia, anemia, hyperbilirubinemia, and coagulopathy, were also associated with worse renal outcomes. Additionally, high illness severity scores such as MELD, APACHE II, and SAPS II reflected systemic compromise and were strong predictors of AKI.

**Table 4:** Documented risk factors for aki after lt.

Author	Year	Documented risk factor
Chiu et al^15^	2023	High levels of total bilirubin (7.7 mg/dL ± 7), prolonged activated partial thromboplastin time (39.1 seconds ± 11), elevated C-reactive protein (1.5 mg/dL ± 3.4), use of certain medications, such as non-steroidal anti-inflammatory drugs (18.1%) and angiotensin-converting enzyme inhibitors (5.8%), comorbidities such as diabetes mellitus (15.2%), hypertension (9.5%) and liver cirrhosis (48.6%).
Rana et al^16^	2023	Male gender (29%), history of hepatorenal syndrome (23.7%), intraoperative use of inotropes (23,7%), MELD score >14 (20.3%), preoperative proteinuria > 0.5 g/day (42.3%), and estimated GFR <90 ml/min/1.73 m^2^ (30.5%).
Cai et al^17^	2023	Youngest age of transplant recipients (44.83 years ± 10.88), lower body mass index (BMI) (22.86 Kg/m^2^ ± 2.94) higher preoperative serum creatinine levels (8.6 mg/dL ± 4.38), lower preoperative estimated glomerular filtration rate eGFR (110.63 ml/min/1.73 m^2^ ± 38.23), increased intraoperative blood loss (2335.71 mL ± 1946.55), higher levels of aspartate aminotransferase (AST) in donors (110.63 U/L ± 191.19).
Wu et al^18^	2023	Prolonged operative time (8.02 hours ± 1.2), intraoperative hypotension (57.9%), postoperative shock (26.3%), high cystatin C levels after surgery, blood product transfusions (1600 ml; 800-2400), prolonged an-hepatic phase (69 minutes ± 13).
de la Fuente et al^19^	2022	Scores for MELD (14; 10-19), Acute Physiology and Chronic Health Evaluation II (APACHE II) and Simplified Acute Physiology Score 2 (14; 10.25-17).
Berkowitz et al^20^	2022	Higher BMI (30.3 Kg/m^2^ ± 6.6), preexisting anemia, arrhythmias, fluid and electrolyte disturbances (70.2%), in addition to higher GFR (87.3 ml/min/1.73 m^2^ ± 42.9) and lower MELD score (18 ± 7).
Chan et al^21^	2022	Hypoalbuminemia (3 mg/dL ±0.5), pre-existing Hepatocellular carcinoma (48%), intraoperative blood loss (6070 mL ± 9706)
Fiorelli et al^22^	2022	Intraoperative need for blood transfusion (92%), use of active vessel drug (73%), surgical (31%) and cardiovascular (9.5%) complications and reperfusion syndrome (44%).
Catalán et al^23^	2022	Male gender (64.7%), hepatic encephalopathy (70.6%), high lactate concentration during surgery (7.2 mmol/L ± 3.3).
Chen et al^25^	2021	MELD score < 25 (86%) preoperative anemia (10.19 mg/dL ± 24.2) and hypoalbuminemia (61.9%)
Zhang et al^26^	2021	BMI > 27 kg/m^2^ (47.7%); Hepatic encephalopathy (23%), preoperative serum creatinine > 9 mg/dL (30.9%); duration of clamping of the vena cava > 56 minutes (82.3%); postoperative lactate > 4 mmol/L (68%); peak postoperative AST > 3000 U/L (32.7%).
Ren et al^28^	2020	Preoperative encephalopathy (8.3%), MELD score (18; 11.25-25.75), intraoperative bleeding volume (2.500 mL; 1.950-5.175), blood transfusion volume (7.5 U; 3.75-12), long operation time (442.5 minutes; 393-505.25) and cold ischemia time (415 minutes; 352.5-477.75) and plasma transfusion (1.750 mL; 1.200-2.575).
Guo et al^31^	2020	Preoperative prothrombin time (17.9 seconds; 14.65-23.7); international normalized ratio of prothrombin time (1.51; 1.25-1.98); preoperative total bilirubin (9.5 mg/dL; 2.7-3.76); operative time, total fluid intake (8237.40 mL ± 1926.99); fresh frozen plasma (900 mL; 600-1200); and estimated blood loss (1500 mL; 800-2750)
Tan et al^12^	2019	**Hepatic Ischemia-Reperfusion Injury (HIRI)**: Moderate or severe HIRI, indicated by peak aspartate aminotransferase (AST) levels within 24 hours. Median AST was 2059 U/L in AKI patients versus 1248 U/L in non-AKI patients **Transfusion Requirements**: AKI patients received a median of 4 units of red blood cells (RBC), versus 1 unit for non-AKI patients. Fresh frozen plasma (FFP) units were significantly higher in AKI patients **Intraoperative Blood Loss**: Increased blood loss, reflected in higher volumes of cell salvage blood transfused, was associated with AKI (median 764 mL in AKI vs. 281 mL in non-AKI). **Surgical Technique**: Use of the caval replacement technique showed a higher incidence of AKI than the piggyback technique **Pre-Transplant Renal Impairment**: Although preoperative renal impairment (high serum creatinine) was more common in AKI patients **Combined Impact of HIRI and AKI on Outcomes:** Patients with both AKI and moderate/severe HIRI had longer ICU stays (5 days vs. 2.5 days in non-AKI, delayed extubation (37 hours vs. 16 hours), and lower 90-day survival rates (89% vs. 100%)

Abbreviations – MELD: Model for End-Stage Liver Disease; GFR: Glomerular filtration rate; BMI: Body mass index; APACHE II: Acute Physiology and Chronic Health Evaluation II; DPP: Diastolic Perfusion Pressure; CVP: Central Venous Pressure; CIT: cold ischemia time; WIT: Warm ischemia time; PRBC: packed red blood cells; HIRI: Hepatic Ischemia-Reperfusion Injury; AST: aspartate aminotransferase; RBC: red blood cells; FFP: Fresh frozen plasma.

In contrast, several intraoperative factors are modifiable and therefore critical targets for AKI prevention. Hemodynamic instability, particularly hypotension and the use of vasopressors during surgery, seems to be a consistent and significant contributor to postoperative AKI. Surgical variables such as longer operative duration, prolonged anhepatic phase, and the use of caval replacement (as opposed to the piggyback technique) were also associated with increased risk. Excessive blood loss and need for transfusion, including red blood cells and plasma, further compounded the burden. Moreover, ischemia-reperfusion injury, often indicated by elevated postoperative AST levels, was not only predictive of AKI but also of poorer overall outcomes. Elevated intraoperative lactate and postoperative cystatin C levels further reflect systemic stress and renal compromise.

## Discussion

### Incidence of Aki After Liver Transplantation

In this meta-analysis, we evaluated the incidence of AKI after LT surgery and the use of dialysis for this complication. Significant variations were observed in the included studies in terms of study population, study design, and gender distribution.

Most included studies adopted the KDIGO definition of AKI, while a minority applied AKIN or RIFLE criteria. To address potential bias arising from these variations, we performed a sensitivity analysis restricted to studies using KDIGO criteria. The resulting incidence range (24-84%) was consistent with our main analysis, supporting the robustness of our pooled estimate and reinforcing KDIGO’s utility in detecting early or mild AKI cases in liver transplant recipients.

This criterion is suggested in the literature as the most sensitive, capturing milder or early cases of kidney injury that might be overlooked with less sensitive criteria^13,14^.

The estimated incidence of AKI after LT of 46% is a relatively high rate. An earlier meta-analysis involving 28,844 liver transplant patients identified an AKI incidence of 37.5%, demonstrating an increase in this postoperative complication^4^. Similarly, Thongprayoon et al.^3^ reported an incidence of 40.7% in a meta-analysis of 38 studies. These data suggest that AKI, which involves various pre-, peri-, and postoperative aspects, remains a significant issue, with some cases exceeding 50% incidence^15^. Our study reinforces that this complication is increasingly documented in the literature and highly associated with clinical and surgical factors. Although only more recent articles were included, it is evident that post-LT AKI is an ongoing concern. Of the 30 studies included^12,16,17,18,19,20,21,22,23,24,25,26,27,28,29,30,31,32,33,34,35,36,37,38,39,40,41,42,43,44^ in the past five years, six were published in the last year^16^,^17^,^18^,^19^,^20^,^21^, highlighting the need for future studies to further elucidate the mechanisms and parameters involved in these repercussions.

### Risk Factors for Aki

Variability in demographic characteristics was observed, with mean patient ages ranging from 45 to 58 years and a predominance of male recipients. This finding aligns with Zarbock et al.^13^, who reported a higher incidence of AKI among male patients (67.4%), potentially reflecting hormonal and physiological differences that influence the response to surgical stress and the kidney function. Both younger and older patients were affected by AKI; however, some studies, such as that by Cai et al.^20^, identified younger age as a specific risk factor, possibly due to a more pronounced inflammatory response and increased graft rejection risk in younger individuals.

### Dialysis Requirement After Liver Transplantation

Dialysis rates among AKI patients also varied significantly. Some authors highlighted a substantial need for dialysis, such as in the studies by Cywinski et al.^16^ and Sáez de la Fuente et al.^22^ (52 and 51%, respectively). These variations underscore the need for further analysis of specific risk factors for post-LT AKI development, considering both demographic characteristics and clinical/therapeutic factors.

The 10% incidence of post-surgery dialysis requirement was 2.3% higher than the meta-analysis by Thongprayoon et al.^3^. The increased need for dialysis therapy in patients without previous kidney diseases is significant. This indicates that a subset of patients evolves to severe forms of AKI requiring intervention. Studies suggest that early initiation of post-surgery renal replacement therapy (RRT) prevents metabolic disorders and the progression of AKI to advanced stages^45,46,47,48,49^. Research on the incidence of RRT is crucial for monitoring clinical progression, adjusting therapeutic interventions, and ensuring appropriate continuity of care for postoperative renal complications.

### Methodological Considerations

This study has some limitations that should be considered when interpreting the results. Firstly, despite the methodological rigor employed in the selection of studies, it was not possible to systematically extract data related to follow-up duration, LT modality (such as living versus deceased donor), and outcome frequency. In addition, specific information about the dialysis treatment implemented in the various studies could not be retrieved, as several articles lacked this information in a standardized or complete manner.

High statistical heterogeneity for both AKI (I^2^ = 97%) and dialysis incidence (I^2^ = 95%) was observed across studies. This variability likely reflects differences in study populations, perioperative practices, and AKI definitions. Subgroup analysis or meta-regression to identify sources of heterogeneity were precluded because of the lack of consistently reported variables across studies. Future studies with standard reporting are needed to allow for more detailed stratification.

The studies included in this meta-analysis were conducted across diverse geographic regions, including Asia, Europe, North America, and South America, which provides a high external validity to our findings. However, differences in regional clinical practices, healthcare infrastructure, and patient populations may influence the applicability of results in specific local contexts and contribute to observed heterogeneity.

### Final Considerations

The high incidence of AKI and dialysis requirement after LT underscores the need for vigilant perioperative management and targeted preventive strategies. Enhanced reporting standards and methodological consistency in future research will be crucial to advance the knowledge of this evolving field and ultimately improve patient outcomes.

## Conclusion

The results of our meta-analysis suggest that AKI and dialysis requirements are significant complications after LT. The analysis highlights multiple risk factors, including HTN, diabetes, hyperlactatemia, and prolonged hospitalization, which can guide prevention and management strategies. Despite advancements in surgical techniques and postoperative care, the high incidence of AKI underscores the need for standardized definitions, early identification of at-risk patients, and multidisciplinary approaches to improve outcomes.

## Data Availability

The complete dataset supporting the findings of this study has been made available on SciELO Data and can be accessed at: https://doi.org/10.48331/SCIELODATA.YGDKL8.

## Supplementary Material

The following online material is available for this article:

Table S1 – Risk Bias Assessment.
