# Supplementary material for: Frequency of acute kidney injury in post-liver transplantation and associated factors: a systematic review
Source: J Bras Nefrol. 2025 Oct 10;47(4):e20250022. doi: 10.1590/2175-8239-JBN-2025-0022en (PMC12513560; doi:10.1590/2175-8239-JBN-2025-0022en)
Supplement: Supplementary file 1 [file 2175-8239-jbn-47-4-e20250022-suppl.pdf]

Braz. J. Nephrol.
https://doi.org/10.1590/2175-8239-JBN-2025-0022en

Supplementary Material to "Frequency of acute kidney injury in post-liver transplantation and associated factors: a systematic"

TABLE S1 Risk Bias Assessment (Newcastle-Ottawa Quality Assessment Form For Cohort Studies was used to analyze the methodological quality of the included studies).

		**Items**								
**Autor**	**Ano**	**Selection**				**Comparability**	**Outcome**			
		1	2	3	4	1	1	2	3	**Score**
Cywinski et al	2024	*	*	*	*	**	*	*	*	9/9
Caragata et al	2023	*	*	*	*	*	*	*	*	8/9
Chiu et al	2023	*	*	*	*	*	*	*	*	9/9
Rana et al	2023	*	*	*	*	*	*	*	*	8/9
Cai et al	2023	*	*	*	*	*	*	*	*	8/9
Wu et al	2023	*	*	*	*	*	*	*	*	9/9
de la Fuente et al	2022	*	*	*	*	*	*	*	*	9/9
Berkowitz et al	2022	*	*	*	*	*	*	*	*	8/9
Chan et al	2022	*	*	*	*	**	*	*	*	9/9
Fiorelli et al	2022	*	*	*	*	**	*	*	*	9/9
Catalán et al	2022	*	*	*	*	**	*	*	*	9/9
Park et al	2021	*	*	*	*	**	*	*	*	9/9
Chen et al	2021	*	*		*	*	*	*	*	7/9
Zhang et al	2021	*	*	*	*	**	*	*	*	9/9
Park et al	2020	*	*	*	*	**	*	*	*	9/9
Ren et al	2020	*	*	*	*	**	*	*	*	9/9
Mrzljak et al	2020	*	*	*	*	*	*	*	*	8/9
Savier et al	2020	*	*		*	**	*	*		7/9
Guo et al	2020	*	*	*	*	**	*	*	*	9/9
Shih et l	2020	*	*	*	*	**	*	*	*	9/9
Carrier et al	2020	*	*	*	*	**	*	*	*	9/9
Lee et al	2020	*	*	*	*	*	*	*	*	8/9
Kim et al	2020	*	*	*	*	*	*	*	*	8/9
Feldkamp et al	2020	*	*	*	*	**	*	*	*	9/9
Min et al	2020	*	*	*	*	*	*	*	*	8/9
Kim et al	2019	*	*	*	*	**	*	*	*	9/9
Tan et al	2019	*	*	*	*	**	*	*	*	9/9
Arani et al	2021	*	*	*	*	**	*	*	*	9/9
Neves et al	2022	*	*	*	*	**	*	*	*	9/9
Bao et al	2021	*	*	*	*	*	*	*	*	8/9
