# Supplementary material for: Frequency of acute kidney injury in post-liver transplantation and associated factors: a systematic review
Source: J Bras Nefrol. 2025 Oct 10;47(4):e20250022. doi: 10.1590/2175-8239-JBN-2025-0022en (PMC12513560; doi:10.1590/2175-8239-JBN-2025-0022en)
Supplement: Translation

# Translation: Frequência de injúria renal aguda no pós-transplante hepático e fatores associados: uma revisão sistemática

**DOI:** 10.1590/2175-8239-JBN-2025-0022pt

**Authors:** Ana Flavia Moura, Alessandra Lima Costa, Maria Theresa Corrêa Evangelista, Ana Clara de Lemos Guimarães, Arthur Guimarães de Freitas, Gabriel Pla Cid Vinhaes, Maria Eduarda Serravalle Mata Pires Fernandes, Daniela de Queiroz Moura-Landim, José A. Moura-Neto, Constança Margarida Sampaio Cruz

**Affiliations:** 1 Escola Bahiana de Medicina e Saúde Pública, Departamento de Medicina Interna, Salvador, BA, Brasil.; 2 Universidade Federal da Bahia, Salvador, BA, Brasil.; 3 Universidade de Salvador, Salvador, BA, Brasil.; 4 Hospital Santo Antônio – Obras Sociais Irmã Dulce, Salvador, BA, Brasil.

**Keywords:** Injúria Renal Aguda, Insuficiência Renal, Transplante Hepático, Revisão Sistemática

## Abstract

**Introdução:**

Injúria renal aguda (IRA) é uma complicação comum após o transplante hepático (TH). Está associada a fatores como instabilidade hemodinâmica perioperatória, cirurgia prolongada e uso de imunossupressores nefrotóxicos, contribuindo para mortalidade aumentada, falha do enxerto e permanência hospitalar prolongada.

**Métodos:**

Realizou-se uma busca sistemática nas bases de dados *PubMed, Embase e Cochrane Central Register of Controlled Trials* para identificar estudos observacionais com amostras de, no mínimo, 50 pacientes com 18 anos ou mais submetidos ao TH, analisando a incidência de IRA pós-procedimento e avaliando desfechos renais a longo prazo.

**Resultados:**

Foram incluídos 30 estudos, totalizando 13.653 pacientes. A incidência de IRA pós-TH foi de 46% (IC95%: 45%–47%), com variação significativa entre os estudos (24% a 84%) e alta heterogeneidade (I^2^ = 97%; p < 0,001). A incidência combinada da necessidade de diálise pós-TH foi de 10% (IC95%: 9%–11%), também altamente variável entre os estudos (2% a 36%) com elevada heterogeneidade (I^2^ = 95%; p < 0,001). As complicações pós-operatórias comuns incluíram ventilação mecânica prolongada, disfunção do enxerto, infecções e hipertensão (HTN). Além disso, a análise destacou fatores de risco significativos para IRA, como HTN, diabetes, hiperlactatemia, hiperbilirrubinemia e hospitalização prolongada.

**Conclusão:**

A IRA e a necessidade de diálise são complicações frequentes após o TH. Diversos fatores de risco, incluindo HTN, diabetes e hospitalização prolongada, estão associados a um risco aumentado de IRA pós-TH. A alta incidência de IRA ressalta a importância da identificação precoce de pacientes em risco e de abordagens multidisciplinares para melhorar os desfechos.

## Introdução

A injúria renal aguda (IRA) é uma síndrome clínica caracterizada por um rápido declínio na taxa de filtração glomerular, levando ao acúmulo de produtos metabólicos e a um risco aumentado de mortalidade, eventos cardiovasculares e progressão para doença renal crônica (DRC)^1,2^. A incidência de IRA é particularmente elevada em pacientes submetidos a transplante hepático (TH), afetando 30% a 40% dos receptores na primeira semana pós-operatória. Essa condição é influenciada por diversos fatores, incluindo condições pré-operatórias, instabilidade hemodinâmica perioperatória, tempo cirúrgico prolongado e uso de medicamentos imunossupressores nefrotóxicos^3–5^.

A IRA é uma complicação comum e preocupante no período pós-operatório do TH devido à sua associação com mau prognóstico. Meta-análises anteriores demonstraram que a IRA após o TH está associada a maior mortalidade, rejeição do enxerto e progressão da DRC. Os fatores de risco identificados incluem o uso de vasopressores, transfusões sanguíneas e disfunção hepática pré-existente. No entanto, esses estudos carecem de dados mais recentes, limitando as evidências atuais sobre o tema^3,6^. Além disso, a IRA prolonga a hospitalização e aumenta os custos do tratamento, tornando seu manejo uma prioridade^7–9^. Dessa forma, uma abordagem multidisciplinar e preventiva pode reduzir significativamente as complicações e melhorar o prognóstico dos pacientes submetidos ao transplante hepático.

A complexidade da associação entre TH e função renal ressalta a necessidade de um manejo cuidadoso desses pacientes. De acordo com as diretrizes recentes para IRA, tanto o *Kidney Disease: Improving Global Outcomes* (KDIGO)^10^ quanto a *International Society of Nephrology* (ISN)^11^ enfatizam a importância da identificação precoce do risco e da estratificação para IRA, particularmente em pacientes submetidos a cirurgias de grande porte, com sepse ou expostos a nefrotoxinas. As estratégias preventivas incluem garantir uma hidratação adequada com terapia volêmica direcionada a metas, evitando tanto a hipovolemia quanto a sobrecarga hídrica e minimizando o uso de agentes nefrotóxicos, como aminoglicosídeos, anti-inflamatórios não esteroides e contrastes iodados^10,11^. O manejo e a prevenção, aliados aos avanços nas técnicas cirúrgicas e nos cuidados intensivos, têm o potencial de reduzir significativamente as complicações e melhorar os desfechos de longo prazo para os receptores de transplantes^10,12^.

Diante desse contexto, o objetivo desta meta-análise é compilar dados detalhados de estudos para avaliar a incidência de IRA pós-TH e analisar os desfechos renais a longo prazo, como a progressão da DRC e a dependência de diálise. Com isso, será possível otimizar as estratégias de prevenção e tratamento, melhorando assim os desfechos clínicos e a qualidade de vida dos pacientes transplantados.

## Método

### Desenho do Estudo

Esta é uma revisão sistemática e meta-análise que examina a incidência de IRA após cirurgia de TH e fatores associados, incluindo distúrbios relacionados, necessidade de tratamento dialítico e idade dos pacientes. O estudo foi registrado na plataforma PROSPERO (*International Prospective Register of Systematic Reviews*) sob o código CRD42024583773.

### Estratégia de Busca

Foi realizada uma busca abrangente da literatura, em português e inglês, incluindo todos os estudos disponíveis até julho de 2024. As buscas foram conduzidas nas bases de dados *PubMed*, *Cochrane* e *Embase*, utilizando descritores relacionados à saúde provenientes das plataformas *DeCS*/*MeSH*, combinados com os operadores booleanos “OR” e “AND” para contemplar a população de interesse. Especificamente, foram empregados os seguintes termos: “(*LT* OR *OLT* OR *liver transplantation* OR *Grafting, Liver* OR *Liver Grafting* OR *Transplantation, Liver OR Liver Transplantations* OR *Liver Transplant* OR *Liver Transplants* OR *Transplant, Liver* OR *Hepatic Transplantation* OR *Hepatic Transplantations* OR *Transplantation, Hepatic*) AND (*AKI* OR *acute kidney injury* OR *Acute Kidney Injuries* OR *Kidney Injuries, Acute* OR *Kidney Injury, Acute* OR *Acute Renal Injury* OR *Acute Renal Injuries* OR *Renal Injuries, Acute* OR *Renal Injury, Acute* OR *Renal Insufficiency, Acute* OR *Acute Renal Insufficiencies* OR *Acute Kidney Failure*)”.

### Critérios de Inclusão

A revisão incluiu estudos observacionais prospectivos e retrospectivos com uma amostra de, ao menos, 50 pacientes com idade mínima de 18 anos submetidos à cirurgia de TH, que analisaram a incidência de IRA após o procedimento.

### Critérios de Exclusão

Foram excluídos os estudos que envolviam populações com menos de 18 anos de idade, casos crônicos ou pré-existentes de IRA, revisões (narrativas, de escopo, integrativas ou sistemáticas), relatos/séries de casos, resumos de conferências, dados incompletos ou sobrepostos, ou que não estivessem em português ou inglês. Estudos intervencionais também foram excluídos.

### Seleção e Extração de Dados

Os artigos foram identificados com base na estratégia de busca e importados para o software *Rayyan*. As entradas duplicadas foram removidas por meio do software e de verificações cruzadas manuais. A seleção dos artigos foi realizada manualmente por dois pesquisadores independentes, de forma cega; as discrepâncias foram resolvidas por um terceiro investigador. Os estudos selecionados foram organizados no Microsoft Excel, onde foram extraídos dados como ano de publicação, país, características da amostra (tamanho, idade e sexo), incidência de IRA pós-TH, definições de IRA, principais desfechos, risco de viés, necessidade de diálise e fatores de risco associados.

### Avaliação da Qualidade

A qualidade metodológica dos estudos incluídos foi avaliada utilizando a escala *Newcastle-Ottawa Quality Assessment Form for Cohort Studies*.

### Revisão Sistemática e Meta-Análise

Foi realizada uma revisão sistemática da literatura, seguida por uma meta-análise da incidência de IRA e da necessidade de tratamento dialítico pós-TH por meio de interpolação de dados.

## Resultados

### Seleção e Caracterização dos Estudos Selecionados

A partir da busca sistemática e da aplicação dos critérios de inclusão e exclusão, foram incluídos 30 artigos, envolvendo um total de 13.653 pacientes. O fluxograma PRISMA é apresentado na Figura 1 e o risco de viés na Tabela Suplementar S1. A maioria dos estudos apresentou qualidade de evidência alta ou moderada, conforme avaliado pela escala *Newcastle-Ottawa Quality Assessment Form for Cohort Studies*.

**Figura 1:** Diagrama de fluxo PRISMA 2020 para revisões sistemáticas, que incluiu somente buscas em bancos de dados e registros.

Os estudos incluídos foram publicados entre 2019 e 2024. Do número total de pacientes, 6.122 desenvolveram IRA após cirurgia de TH. Todos os estudos incluídos foram estudos observacionais prospectivos ou retrospectivos publicados em inglês, sendo um estudo (3,2%) do tipo caso-controle (Tabela 1). A maioria dos estudos utilizou as diretrizes KDIGO para a definição de IRA, exceto cinco estudos (16%) que utilizaram as definições RIFLE ou AKIN (Tabela 2). As Tabelas 1 e 2 resumem as características de cada estudo individual.

**Tabela 1:** Características gerais dos estudos incluídos

Autor	Ano	País	Desenho do Estudo	Tamanho da Amostra	Idade	Sexo Feminino
Cywinski et al^13^	2024	EUA	Retrospectivo	1576	56 ± 11	33%
Caragata et al^14^	2023	Canadá	Retrospectivo	1292	58 ± 18	68%
Chiu et al^15^	2023	Taiwan	Retrospectivo	392	54 ± 8	23,7%
Rana et al^16^	2023	Índia	Prospectivo	186	não disponível	68%
Cai et al^17^	2023	China	Retrospectivo	214	45 ± 11	13%
Wu et al^18^	2023	China	Prospectivo	120	53 ± 10	20%
de la Fuente et al^19^	2022	Espanha	Prospectivo	150	55,6 (51-63)	23%
Berkowitz et al^20^	2022	EUA	Retrospectivo	598	54 ± 11	34%
Chan et al^21^	2022	Taiwan	Retrospectivo	512	54 ± 8	26%
Fiorelli et al^22^	2022	Itália	Prospectivo	1681	53	25%
Catalán et al^23^	2022	México	Retrospectivo	86	52 (18-69)	53%
Park et al^24^	2021	Coreia do Sul	Retrospectivo	591	53 ± 9	70%
Chen et al^25^	2021	China	Retrospectivo	1505	49 ±10	18%
Zhang et al^26^	2021		Retrospectivo	204	49	20%
Park et al^27^	2020	Coreia do Sul	Retrospectivo	479	53 (48-59)	31%
Ren et al^28^	2020	China	Retrospectivo	173	51 ± 9	8%
Mrzljak et al^29^	2020	Croácia	Retrospectivo	205	57 ±10	26%
Savier et al^30^	2020	França	Retrospectivo	159	50	não disponível
Guo et al^31^	2020	China	Caso-Controle	122	51 ± 11	20%
Shih et al^32^	2020		Retrospectivo	60	55 ± 6	não disponível
Carrier et al^33^	2020	Canadá	Retrospectivo	524	52 ± 11	33%
Lee et al^34^	2020	Coreia do Sul	Retrospectivo	294	52,7	53%
Kim et al^35^	2020	Coreia do Sul	Retrospectivo	676	54,7	28%
Feldkamp et al^36^	2020	Alemanha	Retrospectivo	149	48,6	37%
Min et al^37^	2020	Coreia do Sul	Retrospectivo	423	51,5	26%
Kim et al^38^	2019	Coreia do Sul	Retrospectivo	734	53 (48-58)	24%
Tan et al^12^	2019	China	Retrospectivo	227	46 ± 10	19%
Arani et al^39^	2021	Irã	Retrospectivo	173	45 ± 17	45%
Neves et al^40^	2022	Brasil	Retrospectivo	49	54 (43-65)	39%
Bao et al^41^	2021	China	Retrospectivo	99	49 ± 10	8%

Abreviaturas – Fem: Feminino. As variáveis categóricas e numéricas foram descritas utilizando média e desvio padrão; mediana e intervalo interquartil; porcentagem válida ou valor absoluto.

**Tabela 2:** Características específicas dos estudos incluídos

Autor	Ano	Definição de IRA	Incidência de IRA	Idade - IRA	IRA – sexo fem.	Diálise
Cywinski et al^13^	2024	KDIGO	73,6%	não disponível	não disponível	não disponível
Caragata et al^14^	2023	KDIGO	40%	não disponível	não disponível	52
Chiu et al^15^	2023	KDIGO	62%	54 ± 8	25,3%	17
Rana et al^16^	2023	ni	31,7%	51 (41-57)	5,1%	25
Cai et al^17^	2023	KDIGO	49%	48 ± 10	11,4%	10
Wu et al^18^	2023	KDIGO	48,3%	53 (11)	22,4%	8
de la Fuente et al^19^	2022	KDIGO	58,6%	58 (51-65)	22%	51
Berkowitz et al^20^	2022	KDIGO	42,6%	52	33%	não disponível
Chan et al^21^	2022	KDIGO	35%	55 ± 8	27%	não disponível
Fiorelli et al^22^	2022	RIFLE	21,8%	54	26%	não disponível
Catalán et al^23^	2022	KDIGO	39,5%	51 ± 11	35,2%	9
Park et al^24^	2021	KDIGO	25,2%	53 (48–59)	23%	35
Chen et al^25^	2021	KDIGO	44,5%	49 ± 10	19%	não disponível
Zhang et al^26^	2021	KDIGO	55,3%	49	12%	não disponível
Park et al^27^	2020	KDIGO	24%	52 (47-59)	27%	não disponível
Ren et al^28^	2020	KDIGO	27,7%	52 ± 10	10%	23
Mrzljak et al^29^	2020	KDIGO	45,3%	56 ± 9	22,6%	5
Savier et al^30^	2020	KDIGO	29%	não disponível	não disponível	não disponível
Guo et al^31^	2020	KDIGO	42,6%	53 ± 11	29%	12
Shih et al^32^	2020	ni	41,6%	não disponível	não disponível	não disponível
Carrier et al^33^	2020	ni	69,8%	não disponível	não disponível	31
Lee et al^34^	2020	AKIN	25,8%	55	72%	não disponível
Kim et al^35^	2020	KDIGO	40,6%	não disponível	não disponível	não disponível
Feldkamp et al^34^	2020	KDIGO/ RIFLE / AKIN	65,7%	47	33%	54
Min et al^36^	2020	AKIN	87,2%	52	29%	não disponível
Kim et al^37^	2019	KDIGO	36,1%	54 (49-59)	26%	não disponível
Tan et al^12^	2019	KDIGO	46,6%	47 ± 10	15%	11
Arani et al^39^	2021	AKIN	68,2%	48 ± 15	46%	25
Neves et al^40^	2022	KDIGO	83,6%	61 (43-65)	22%	não disponível
Bao et al^41^	2021	KDIGO	29,2%	50 ± 8	3,4%	4

Abreviaturas – IRA: Injúria Renal Aguda; KDIGO: Kidney Disease Improving Global Outcomes; AKIN: Acute Kidney Injury Network; RIFLE: Risk Injury Failure Loss End-stage kidney disease. Fem.: Feminino. ni: não informado. As variáveis categóricas e numéricas foram descritas utilizando média e desvio padrão; mediana e intervalo interquartil; porcentagem válida ou valor absoluto.

### Incidência de ira Após o Transplante Hepático

A incidência estimada de IRA após o TH foi de 46% (IC 95%: 45–47%; Figura 2). Foi realizada uma análise de sensibilidade apenas com estudos que utilizaram as definições do KDIGO para IRA.

**Figura 2:** Gráfico de floresta da incidência estimada de IRA após o TH.

### Tratamento Dialítico Para ira Após o Transplante Hepático

Um total de 423 pacientes necessitaram de diálise após o transplante hepático, com incidência de 10% (IC 95%: 9–11%; Figura 3).

**Figura 3:** Gráfico de floresta da incidência estimada de pacientes que necessitaram de diálise após o transplante hepático.

### Distúrbios Documentados

Outros distúrbios e complicações documentados após a cirurgia de TH são apresentados na Tabela 3, incluindo ventilação mecânica prolongada, irregularidades relacionadas ao enxerto, infecções, hipertensão (HTN) e outros.

**Tabela 3:** Outros distúrbios e complicações documentados após cirurgia de transplante hepático.

Autor	Ano	Outros distúrbios documentados
Cywinski et al^13^	2024	Eventos cardiovasculares adversos maiores (ECAM) e disfunção precoce do enxerto (DPE)
Caragata et al^14^	2023	Mortalidade precoce, complicações cardíacas e disfunção precoce do enxerto
Chiu et al^15^	2023	Diabetes e hipertensão
Rana et al^16^	2023	Doença renal crônica
Cai et al^17^	2023	Infecções (pulmonares e sepse), hemorragia gastrointestinal, acidente vascular cerebral e doença do enxerto versus hospedeiro (GVHD)
Wu et al^18^	2023	Infecções pós-operatórias, tempo prolongado em ventilação mecânica, choque pós-operatório e disfunção precoce do enxerto
de la Fuente et al^19^	2022	Duração prolongada da ventilação mecânica, infecções nosocomiais e disfunção precoce do enxerto hepático
Fiorelli et al^22^	2022	Tempo prolongado em ventilação mecânica
Zhang et al^26^	2021	Hiperlactatemia
Savier et al^30^	2020	Complicações biliares
Kim et al^35^	2019	Pressão venosa basal elevada, redução da saturação de oxigênio no sangue venoso e aumento do volume diastólico final do ventrículo direito
Tan et al^12^	2019	Infecções e tempo prolongado de intubação
Neves et al^40^	2022	Necessidade de vasopressores, lactato elevado, transfusão e reintervenção
Bao et al^41^	2021	Hipertensão

### Fatores de Risco Documentados

A Tabela 4 apresenta os fatores de risco para IRA após o TH identificados nos estudos incluídos nesta meta-análise. O risco de desenvolver IRA foi influenciado por uma série de fatores pré-operatórios não modificáveis, incluindo variáveis demográficas, como sexo masculino e, em algumas coortes, idade mais jovem. Condições clínicas como diabetes mellitus, HTN, encefalopatia hepática e carcinoma hepatocelular contribuíram para uma maior suscetibilidade. A insuficiência renal basal (evidenciada por elevação da creatinina, proteinúria ou redução da taxa de filtração glomerular) exacerbou ainda mais o risco. Anormalidades laboratoriais, incluindo hipoalbuminemia, anemia, hiperbilirrubinemia e coagulopatia, também estiveram associadas a piores desfechos renais. Além disso, escores elevados de gravidade da doença, como MELD, APACHE II e SAPS II, refletiram comprometimento sistêmico e foram fortes preditores de IRA.

**Tabela 4:** Fatores de risco documentados para ira após th

Autor	Ano	Fator de risco documentado
Chiu et al^15^	2023	Níveis elevados de bilirrubina total (7,7 mg/dL ± 7), tempo de tromboplastina parcial ativada prolongado (39,1 segundos ± 11), proteína C reativa elevada (1,5 mg/dL ± 3,4), uso de determinados medicamentos, como anti-inflamatórios não esteroides (18,1%) e inibidores da enzima conversora da angiotensina (5,8%), comorbidades como diabetes mellitus (15,2%), hipertensão (9,5%) e cirrose hepática (48,6%).
Rana et al^16^	2023	Sexo masculino (29%), histórico de síndrome hepatorrenal (23,7%), uso intraoperatório de inotrópicos (23,7%), escore MELD >14 (20,3%), proteinúria pré-operatória > 0,5 g/dia (42,3%) e TFG estimada <90 ml/min/1,73 m^2^ (30,5%).
Cai et al^17^	2023	Idade mais jovem dos receptores de transplante (44,83 anos ± 10,88), menor índice de massa corporal (IMC) (22,86 Kg/m^2^ ± 2,94) níveis mais elevados de creatinina sérica pré-operatória (8,6 mg/dL ± 4,38), menor taxa de filtração glomerular estimada pré-operatória (TFGe) (110,63 ml/min/1,73 m^2^ ± 38,23), aumento da perda sanguínea intraoperatória (2335,71 mL ± 1946,55), níveis mais elevados de aspartato aminotransferase (AST) nos doadores (110,63 U/L ± 191,19).
Wu et al^18^	2023	Tempo operatório prolongado (8,02 horas ± 1,2), hipotensão intraoperatória (57,9%), choque pós-operatório (26,3%), níveis elevados de cistatina C após a cirurgia, transfusões de hemocomponentes (1600 ml; 800-2400), fase anepática prolongada (69 minutos ± 13).
de la Fuente et al^19^	2022	Escores para MELD (14; 10-19), Fisiologia Aguda e Avaliação de Saúde Crônica II (APACHE II) e Escore Simplificado de Fisiologia Aguda 2 (14; 10,25-17).
Berkowitz et al^20^	2022	IMC mais elevado (30,3 Kg/m^2^ ± 6,6), anemia pré-existente, arritmias, distúrbios hidroeletrolíticos (70,2%), além de maior TFG (87,3 ml/min/1,73 m^2^ ± 42,9) e menor escore MELD (18 ± 7).
Chan et al^21^	2022	Hipoalbuminemia (3 mg/dL ±0,5), carcinoma hepatocelular pré-existente (48%), perda sanguínea intraoperatória (6070 mL ± 9706).
Fiorelli et al^22^	2022	Necessidade intraoperatória de transfusão sanguínea (92%), uso de medicamento vasoativo (73%), complicações cirúrgicas (31%) e cardiovasculares (9,5%) e síndrome de reperfusão (44%).
Catalán et al^23^	2022	Sexo masculino (64,7%), encefalopatia hepática (70,6%), alta concentração de lactato durante a cirurgia (7,2 mmol/L ± 3,3).
Chen et al^25^	2021	Escore MELD < 25 (86%), anemia pré-operatória (10,19 mg/dL ± 24,2) e hipoalbuminemia (61,9%).
Zhang et al^26^	2021	IMC > 27kg/m^2^ (47,7%); encefalopatia hepática (23%), creatinina sérica pré-operatória > 9 mg/dL (30,9%); duração do clampeamento da veia cava > 56 minutos (82,3%); lactato pós-operatório > 4 mmol/L (68%); pico de AST pós-operatório > 3000 U/L (32,7%).
Ren et al^28^	2020	Encefalopatia pré-operatória (8,3%), escore MELD (18; 11,25-25,75), volume de sangramento intraoperatório (2.500 mL; 1.950-5.175), volume de transfusão sanguínea (7,5 U; 3,75-12), tempo de cirurgia prolongado (442,5 minutos; 393-505,25) e tempo de isquemia fria (415 minutos; 352,5-477,75) e transfusão de plasma (1,750 mL; 1,200-2,575).
Guo et al^31^	2020	Tempo de protrombina pré-operatório (17,9 segundos; 14,65-23,7); razão normalizada internacional do tempo de protrombina (1,51; 1,25-1,98); bilirrubina total pré-operatória (9,5 mg/dL; 2,7-3,76); tempo cirúrgico, ingestão total de fluidos (8237,40 mL ± 1926,99); plasma fresco congelado (900 mL; 600-1200); e perda sanguínea estimada (1500 mL; 800-2750).
Tan et al^12^	2019	**Lesão de Isquemia-Reperfusão Hepática (LIRH)**: LIRH moderada ou grave, indicada por níveis de pico de aspartato aminotransferase (AST) dentro de 24 horas. A mediana da AST foi de 2059 U/L em pacientes com IRA versus 1248 U/L em pacientes sem IRA. **Requisitos para Transfusão**: Pacientes com IRA receberam uma mediana de 4 unidades de hemácias (RBC) versus 1 unidade para pacientes sem IRA. O número de unidades de plasma fresco congelado (PFC) foi significativamente maior em pacientes com IRA. **Perda Sanguínea Intraoperatória:** O aumento da perda sanguínea, refletido em volumes mais elevados de sangue recuperado transfundido, foi associado à IRA (mediana de 764 mL na IRA vs. 281 mL na ausência de IRA). **Técnica Cirúrgica:** O uso da técnica de substituição da veia cava apresentou incidência mais elevada de IRA em comparação à técnica piggyback. **Insuficiência Renal Pré-Transplante:** Embora a insuficiência renal pré-operatória (creatinina sérica elevada) fosse mais comum em pacientes com IRA. **Impacto Combinado de LIRH e IRA nos Desfechos:** Pacientes com IRA e LIRH moderada/grave apresentaram maior tempo de permanência na UTI (5 dias vs. 2,5 dias em pacientes sem IRA), extubação tardia (37 horas vs. 16 horas) e menores taxas de sobrevida em 90 dias (89% vs. 100%).

Abreviaturas – MELD: Modelo para Doença Hepática Terminal; TFG: Taxa de Filtração Glomerular; IMC: Índice de Massa Corporal; APACHE II: Fisiologia Aguda e Avaliação de Saúde Crônica II; DPP: Pressão de Perfusão Diastólica; PVC: Pressão Venosa Central; TIF: Tempo de Isquemia Fria; TIQ: Tempo de Isquemia Quente; CH: concentrado de hemácias; LIRH: Lesão de Isquemia-Reperfusão Hepática; AST: aspartato aminotransferase; H: hemácias; PFC: plasma fresco congelado.

Em contrapartida, diversos fatores intraoperatórios são modificáveis e, portanto, alvos críticos para a prevenção da IRA. A instabilidade hemodinâmica, particularmente a hipotensão e o uso de vasopressores durante a cirurgia, parece ser um contribuidor consistente e significativo para a IRA no pós-operatório. Variáveis cirúrgicas, como maior duração do procedimento, fase anepática prolongada e uso de substituição da veia cava (em oposição à técnica *piggyback*), também foram associadas a um risco aumentado. A perda excessiva de sangue e a necessidade de transfusão, incluindo hemácias e plasma, agravam ainda mais o quadro. Além disso, a lesão de isquemia-reperfusão, frequentemente indicada por níveis elevados de AST no pós-operatório, mostrou-se preditiva não somente de IRA, mas também de desfechos globais mais desfavoráveis. Os níveis elevados de lactato intraoperatório e de cistatina C no pós-operatório refletem ainda mais o estresse sistêmico e o comprometimento renal.

## Discussão

### Incidência de ira Após o Transplante Hepático

Nesta meta-análise, avaliamos a incidência de IRA após a cirurgia de TH e o uso de diálise para essa complicação. Foram observadas variações significativas entre os estudos incluídos quanto à população estudada, desenho do estudo e distribuição por sexo.

A maioria dos estudos incluídos adotou a definição de IRA proposta pelo KDIGO, enquanto uma minoria aplicou os critérios AKIN ou RIFLE. Para abordar o potencial viés decorrente dessas variações, realizamos uma análise de sensibilidade restrita aos estudos que utilizaram os critérios KDIGO. A faixa de incidência resultante (24-84%) foi consistente com nossa análise principal, corroborando a robustez de nossa estimativa combinada e reforçando a utilidade do KDIGO na detecção de casos precoces ou leves de IRA em receptores de transplante hepático.

Este critério é sugerido na literatura como o mais sensível, capturando casos mais leves ou precoces de injúria renal que poderiam ser negligenciados por critérios menos sensíveis^13,14^.

A incidência estimada de IRA pós-TH, de 46%, representa uma taxa relativamente elevada. Uma meta-análise anterior envolvendo 28.844 pacientes submetidos a transplante hepático identificou uma incidência de IRA de 37,5%, demonstrando um aumento nessa complicação pós-operatória^4^. De forma semelhante, Thongprayoon et al.^3^ relataram uma incidência de 40,7% em uma meta-análise de 38 estudos. Esses dados sugerem que a IRA, que envolve diversos aspectos pré, peri e pós-operatórios, permanece uma questão significativa, com alguns casos excedendo 50% de incidência^15^. Nosso estudo reforça que essa complicação vem sendo cada vez mais documentada na literatura e está altamente associada a fatores clínicos e cirúrgicos. Embora apenas artigos mais recentes tenham sido incluídos, é evidente que a IRA pós-TH é uma preocupação constante. Dos 30 estudos incluídos^12,16–44^ nos últimos cinco anos, seis foram publicados no último ano^16–21^, destacando a necessidade de estudos futuros para elucidar com mais detalhes os mecanismos e parâmetros envolvidos nessas repercussões.

### Fatores de Risco para ira

Foi observada variabilidade nas características demográficas, com a média de idade dos pacientes variando entre 45 e 58 anos e predominância de receptores do sexo masculino. Esse achado está em consonância com Zarbock et al.^13^, que relataram uma maior incidência de IRA entre pacientes do sexo masculino (67,4%), refletindo potencialmente diferenças hormonais e fisiológicas que influenciam a resposta ao estresse cirúrgico e à função renal. Tanto pacientes mais jovens quanto mais velhos foram afetados pela IRA; entretanto, alguns estudos, como o de Cai et al.^20^, identificaram idade mais jovem como um fator de risco específico, possivelmente em razão de uma resposta inflamatória mais acentuada e maior risco de rejeição do enxerto em indivíduos mais jovens.

### Necessidade de Diálise Após o Transplante Hepático

As taxas de diálise entre pacientes com IRA também variaram significativamente. Alguns autores destacaram uma necessidade substancial de diálise, como nos estudos de Cywinski et al.^16^ e Sáez de la Fuente et al.^22^ (52% e 51%, respectivamente). Essas variações ressaltam a necessidade de uma análise mais aprofundada de fatores de risco específicos para o desenvolvimento de IRA pós-TH, considerando tanto as características demográficas quanto os fatores clínicos/terapêuticos.

A incidência de 10% da necessidade de diálise no pós-operatório foi 2,3% superior à relatada na meta-análise de Thongprayoon et al.^3^. O aumento da necessidade de terapia dialítica em pacientes sem doenças renais prévias é significativo. Isso indica que um subgrupo de pacientes evolui para formas graves de IRA, exigindo intervenção. Estudos sugerem que o início precoce da terapia renal substitutiva (TRS) no pós-operatório previne distúrbios metabólicos e a progressão da IRA para estágios avançados^45–49^. A pesquisa sobre a incidência de TRS é crucial para o monitoramento da progressão clínica, o ajuste das intervenções terapêuticas e a garantia da continuidade adequada dos cuidados para complicações renais pós-operatórias.

### Considerações Metodológicas

Este estudo apresenta algumas limitações que devem ser consideradas na interpretação dos resultados. Em primeiro lugar, apesar do rigor metodológico empregado na seleção dos estudos, não foi possível extrair sistematicamente dados relacionados à duração do acompanhamento, modalidade de TH (por exemplo, doador vivo versus doador falecido) e frequência dos desfechos. Além disso, não foi possível recuperar informações específicas sobre o tratamento dialítico implementado nos diversos estudos, uma vez que vários artigos não apresentavam essas informações de maneira padronizada ou completa.

Foi observada uma elevada heterogeneidade estatística tanto para a IRA (I^2^ = 97%) como para a incidência de diálise (I^2^ = 95%) entre os estudos. Esta variabilidade provavelmente reflete diferenças nas populações dos estudos, nas práticas perioperatórias e nas definições de IRA. A análise de subgrupos ou a metarregressão para identificar as fontes de heterogeneidade foram impossibilitadas devido à falta de variáveis relatadas de forma consistente entre os estudos. São necessários estudos futuros com relatórios padronizados para permitir uma estratificação mais detalhada.

Os estudos incluídos nesta meta-análise foram conduzidos em diversas regiões geográficas, incluindo Ásia, Europa, América do Norte e América do Sul, o que confere uma elevada validade externa aos nossos achados. No entanto, diferenças nas práticas clínicas regionais, infraestrutura de saúde e populações de pacientes podem influenciar a aplicabilidade dos resultados em contextos locais específicos e contribuir para a heterogeneidade observada.

### Considerações Finais

A alta incidência de IRA e necessidade de diálise após o TH ressalta a necessidade de um manejo perioperatório cuidadoso e estratégias de prevenção direcionadas. Padrões de relato aprimorados e consistência metodológica em pesquisas futuras serão cruciais para o avanço do conhecimento neste campo em evolução e, em última instância, para a melhoria dos desfechos dos pacientes.

## Conclusão

Os resultados da nossa meta-análise sugerem que a IRA e a necessidade de diálise constituem complicações significativas após o TH. A análise destaca múltiplos fatores de risco, incluindo HTN, diabetes, hiperlactatemia e hospitalização prolongada, que podem orientar as estratégias de prevenção e manejo. Apesar dos avanços nas técnicas cirúrgicas e nos cuidados pós-operatórios, a elevada incidência de IRA destaca a necessidade de definições padronizadas, identificação precoce de pacientes em risco e abordagens multidisciplinares para melhorar os desfechos.

## Data Availability

O conjunto completo de dados que sustenta os achados deste estudo foi disponibilizado no *SciELO Data* e pode ser acessado em: https://doi.org/10.48331/SCIELODATA.YGDKL8.

## Material Suplementar

O seguinte material online está disponível para este artigo:

Tabela S1 – Avaliação do Risco de Viés.
